# Understanding Apoptosis and Apoptotic Pathways Targeted Cancer Therapeutics

**DOI:** 10.15171/apb.2019.024

**Published:** 2019-06-01

**Authors:** Rehmat Jan, Gul-e-Saba Chaudhry

**Affiliations:** Institute of Marine Biotechnology, Universiti Terengganu Malaysia, 21030 Terengganu, Malaysia.

**Keywords:** Apoptosis, Apoptosis pathways, Cancer, c-FLIP, Caspases, Death receptors, Targeted drugs

## Abstract

Various physiological processes involve appropriate tissue developmental process and homeostasis - the pathogenesis of several diseases connected with deregulatory apoptosis process. Apoptosis plays a crucial role in maintaining a balance between cell death and division, evasion of apoptosis results in the uncontrolled multiplication of cells leading to different diseases such as cancer. Currently, the development of apoptosis targeting anticancer drugs has gained much interest since cell death induced by apoptosis causes minimal inflammation. The understanding of complexities of apoptosis mechanism and how apoptosis is evolved by tumor cells to oppose cell death has focused research into the new strategies designed to induce apoptosis in cancer cells. This review focused on the underlying mechanism of apoptosis and the dysregulation of apoptosis modulators involved in the extrinsic and intrinsic apoptotic pathway, which include death receptors (DRs) proteins, cellular FLICE inhibitory proteins (c-FLIP), anti-apoptotic Bcl-2 proteins, inhibitors of apoptosis proteins (IAPs), tumor suppressor (p53) in cancer cells along with various current clinical approaches aimed to selectively induce apoptosis in cancer cells.

## Introduction

**Figure 1:**
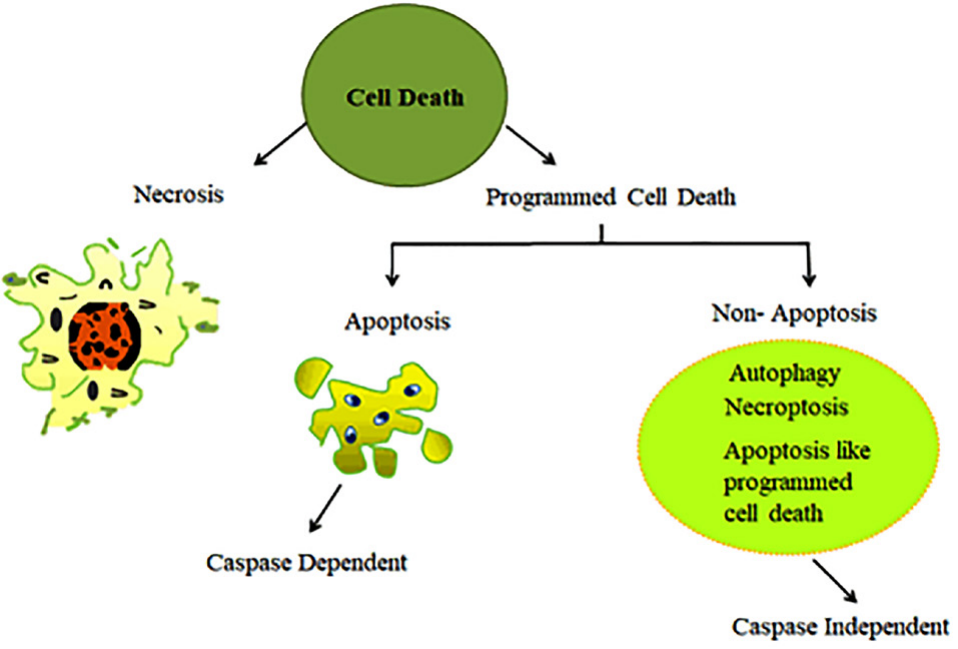
General mode of cancer cell death. Cancer cell death involves two broadly defined mechanisms: programmed cell death and necrosis. Programmed cell death mainly apoptosis and nonapoptosis base cell death, such as autophagy, necroptosis and apoptosis like programmed cell death.

Cell death is an essential process in the development, tissue homeostasis and integrity of multicellular organisms. The cell proliferation and elimination is necessary to maintain a homeostasis physiological processes in the adult organism.^1,2^ The unwanted cells removed during the process of metamorphosis, embryogenesis, pathogenesis as well as tissue turnover.^3,4^ Cell death typically involves two broadly deﬁned mechanisms: programmed cell death and necrosis (Figure 1).^5^ Cell death which includes a genetically programmed process of cell suicide in response to particular signals is called programmed cell death.^6,7^ Usually, programmed cell death controlled by a variety of extracellular and intracellular signals which are directed by the environment of the cell and intracellular signals.^8^ Programmed cell death distinguished from cell necrosis as it has distinct morphological characteristics, maintains tissue homeostasis and regulates the proper number of cells in multicellular organisms by eliminating unwanted cells.^3,9^ Different endogenous tissue-specific agents and exogenous cell-damaging agents initiate programmed cell death in particular cell type under critical physiological conditions.^10^ Exogenous activations of programmed cell death include physical agents and infectious agents that act on most types of cells. Physical agents include radiation, physical trauma, and chemotherapeutic drugs while infectious agents include viruses and bacterial toxins.^11^ Further, internal imbalances such as growth factors withdrawal, ablation of a trophic hormone, treatment with glucocorticoids and loss of matrix attachment can trigger apoptosis.^10^ Although various research groups have often equated programmed cell death with apoptosis, recent studies have proven that non-apoptotic forms of programmed cell death also exist which lacks involvement of the mechanism of apoptosis. Therefore, programmed cell death and apoptosis should never be considered synonymous.^12,13^ Kerr et al proposed the term apoptosis used to describe a morphologically distinct pattern of cell death.^14,15^

Apoptosis extensively described as a significant mechanism of regulated death that occurs not only as a result of cell damage or external stress but it also takes place during normal development, and morphogenesis.^16^ Apoptosis tightly regulated by different groups of the executioner and regulatory molecules. Mechanism of action of apoptotic cell death typically characterized by condensation of chromatin material, fragmentation of DNA occurred in the nucleus, cell shrinkage, dynamic membrane blebbing, and loss of adhesion to extracellular matrices. Further, biochemical alterations include; externalization of phosphatidylserine, and the activation of cysteine aspartyl proteases, called caspases which leads to the cell death.^14,16-20^

Apoptosis typically distinguished from necrosis, which was assumed to represent an opposite way of an unordered cellular explosion in response to severe and irresistible trauma. Interest in non-apoptotic forms of programmed cell death is gradually increasing as more information on this type of cell death is collected.^21^ Non-apoptotic cell death types include autophagy, necroptosis, and apoptosis-like programmed cell death. Autophagy or autophagic cell death termed as type II cell death. Autophagic cell death is a self-degradative process, and it plays a vital role in the degradation of cellular components inside the dying cell in autophagic vacuoles. Autophagy is also known as vacuolar cell death and is very common in the invertebrate tissue.^22,23^ Necroptosis is a programmed form of necrotic death, and it initiated by same death signals that induce apoptosis.^24^ Necroptosis is very common in vivo, in physical traumas, death inflicted by infection and in diverse forms of neurodegeneration. It believed that apoptosis and necroptosis (a regulated and programmed form of cell death) shares several vital processes. Several death receptors (DRs) such as FAS and TNFR that are known to induce apoptosis also induce necroptosis in different cell types.^25^ However, programmed necrosis has been seen only under a specific condition when apoptosis has been chemically or genetically repressed or blocked.^16^ Moreover, another form is apoptosis-like programmed cell death describe the type of cell death which involve apoptotic features, but the cell death occurs in a caspase-independent manner.^26^

Necrosis might happen during several diseases including cancer, neurodegenerative and autoimmune diseases. Necrosis traditionally considered as random, uncontrolled process which is usually initiated by certain stimuli like toxic trauma or physical damage.^27,28^ Necrosis morphologically characterized by the swelling of cytoplasm and organelles (endoplasmic reticulum and mitochondria), the disruption of the plasma membrane leading to the release of cellular components and cell lysis.^16,29^ Cell death by necrosis linked to chronic inflammation, caused by necrosis could enhance the proliferation of tumors.^30^ However, there is another perception that necrosis could also be programmed in nature as well. Programmed necrosis has been seen only under a specific condition in which apoptosis is chemically or genetically repressed or inhibited.^31^ Various anti-apoptotic proteins of the Bcl-2 family have been shown to inhibit both apoptotic and necrotic. Intracellular ATP depletion can switch an apoptotic response to a necrotic one. Hence, apoptosis and necrosis are not necessarily independent pathways. Instead, they may share some common messengers, activators, and inhibitors.^32^

### Understanding apoptosis pathways for cancer therapeutics

**Figure 2:**
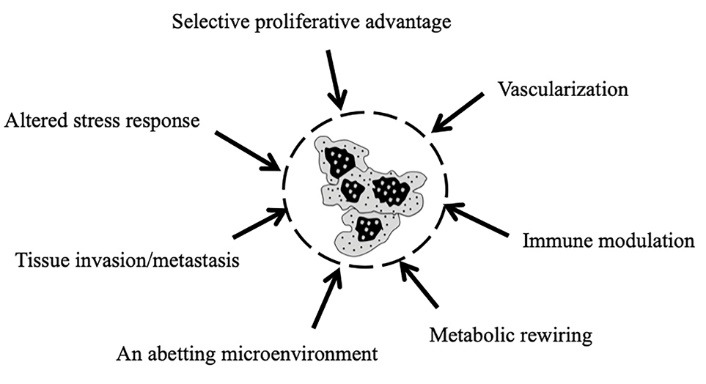
Hallmark of cancer. Various acquire features of cancer cell, include selective proliferative advantage, vascularization, immune modulation, metabolic rewiring, an abetting microenvironment, tissue invasion/metastasis and altered stress response.

The word cancer was first named by a physician Hippocrates around 460-370 B.C. and originated from a Greek word “*karkinos*” which means carcinoma.^33^ Cancer defined as an uncontrolled growth of the cell in an abnormal manner which alters the structure of surrounding tissues.^34^ Cancer cell genotypes demonstrate seven essential alterations in cell physiology which leads to its progression and metastasis.^35^ Figure 2 shows the seven “hallmark of cancer” which contribute towards tumor development. The carcinogenesis comprised of complex multiple steps process where single cell converted to the tumor and move to another site via the process of metastasis. Apoptosis is the vital and crucial mechanism which maintains the balance between survival and death in cells to prevent cancer and other related diseases.^36^

Recent advances in cancer research are focused on the development of new drugs that halt the escape behavior of cancer cells via the execution of apoptosis. To this context, novel apoptotic inducers or sensitizers have been used with the combination of current drugs. Defects in the apoptosis-inducing pathways can eventually lead to the multiplication of neoplastic cells. The resistance of apoptosis stimulates aberrant cellular multiplication which eventually leads to tumorigenesis and is a significant hurdle to active cancer treatment.^37,38^ The induction of apoptosis in cancer cells and limit concurrent death of normal cells is the primary objective of cancer therapy.^39^ Some proteins have been studied to exert pro- and anti-apoptotic activity in the cell and the proportion of these proteins plays an essential part in the regulation of cell death.^40^ Similarly, the induction of cancer apoptosis is among the main approaches in cancer gene therapy or immunotherapy. The apoptotic inducer, mediator or executioner gene is routinely incorporated in cancer cells to reverse the deficiency of its endogenous counterpart.^41,42^ Understanding the cascade of events that trigger apoptosis in response to chemotherapy, and dysfunction of the apoptosis process give insight toward the novel effective therapeutic approach to the development of molecular-targeted specific therapies against cancer.

The mechanism of apoptosis mainly consist of two core pathways involved in inducing apoptosis; extrinsic pathway and intrinsic pathway. Extrinsic pathway refers to DR-mediated pathway, and the intrinsic pathway is a mitochondrial-mediated pathway.^15^ Both of these apoptotic pathways, extrinsic and intrinsic pathways might be lead to same terminal (execution pathway).^15,40^

### Extrinsic pathway

Apoptotic signaling through the extrinsic pathway engaged when extracellular ligands such as TNF (tumor necrosis factor), Fas-L (Fas ligand), and TRAIL (TNF-related apoptosis-inducing ligand) are attached to the extracellular domain of the DR (transmembrane receptors), i.e., the type 1 TNF receptor (TNFR1), Fas (also called CD95/Apo-1) and TRAIL receptors. The order of events involved in the extrinsic phase of apoptosis well characterized by the FasL/FasR and TNF-α/TNFR1 models.^15,43,44^ This triggering of DRs by specific death ligands (DLs) results in the formation of a death-inducing signaling complex (DISC).^12^ This DISC consists of the DD-containing Fas-associated death domain (DD) as an adaptor molecule, procaspase-8, procaspase-10, and the cellular FLICE inhibitory proteins (c-FLIPs). The caspase 8 activate in such a manner that prodomain of caspase 8 remains at the DISC, while active caspase 8 dissociates from the DISC to start the cascade of caspase activation which constitutes the execution phase of apoptosis.^45^ Experimental evidence shows the excessive role of caspases in apoptosis.^46,47^ Caspases are essential initiators and executioners of the apoptosis, and their function is very closely related to its structure having different substrate preferences. Some caspases have long pro-domains which involve particular motif like the death effector domain (DED), and caspase recruitment domains (CARD), which allow interacting with other proteins, and connect with signaling pathways. DED includes caspase-8 and caspase-10 while CARD involves caspase-1, caspase-2, caspase-4, caspase-5, caspase-9, caspase-11 and caspase-12.^48^ Caspases traditionally classified as initiator and effector or executioner caspases by their position in apoptotic signaling cascades.

### Intrinsic pathway

**Figure 3:**
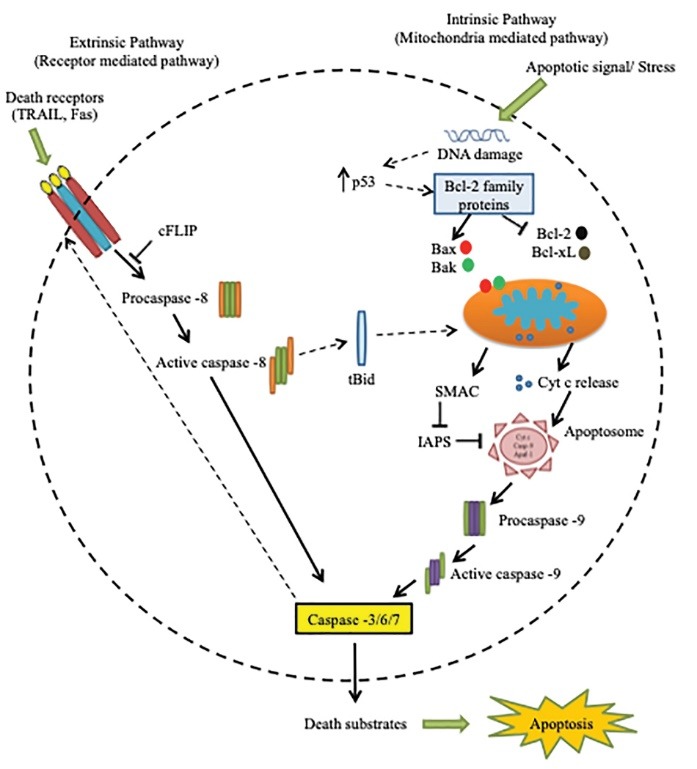
Pathways of Apoptosis. Apoptosis mainly consist of two main pathways and third is executioner pathway of apoptosis. Extrinsic pathway triggered by external stimuli or ligand molecule and particularly involve death receptors (DRs). The intrinsic pathway is mediated by Bax/Bak insertion into mitochondrial membrane, and subsequently, cytochrome c released which combines with Apaf–1 and procaspase-9 to produce apoptosome followed by the activation of caspase 3 cascade of apoptosis. TNF related apoptosis inducing ligand (TRAIL), cellular FLICE inhibitory proteins (cFLIP), Truncated bid (tBid), B-cell lymphoma protein 2 (Bcl-2), Bcl-2 homologue splice variants (Bcl-xL), Cytochrome (Cyt C), Second mitochondrial activator of caspases (SMAC), Inhibitor of apoptosis proteins (IAPs).

The intrinsic pathway refers to mainly mitochondrial-mediated apoptotic pathway. The intrinsic pathway triggered by various extra and intra-cellular stresses, which include oxidative stress, irradiation, and treatment with cytotoxic drugs.^49,50^ Figure 3 shows the pathways of apoptosis, the intrinsic pathway is mediated by Bax/Bak insertion into mitochondrial membrane, and subsequently, cytochrome c released from the mitochondrial intermembrane space into the cytosol.^51^ Bcl-2 and Bcl-xL (Bcl-2 family member) are anti-apoptotic proteins which prevent the release of cytochrome c.^52^ The cytochrome c combines with Apaf–1 and procaspase-9 to produce apoptosome. Apoptosome is a multi-protein complex which comprised of a seven-spoke ring-shaped complex, which triggers caspase 9 followed by the activation of caspase-3 signaling caspase cascade which leads to the demolition of cells and ends up to apoptosis.^43,46,53^ Proteins that are generally involved in intrinsic pathway include SMAC/DIABLO (Second mitochondrial activator of caspases/direct IAP binding protein with low PI), Caspase-9 (Cysteinyl aspartic acid-protease-9), Bcl-2 (B-cell lymphoma protein 2), Bcl-w (Bcl-2 like protein), Nox (Phorbol-12-myristate-13-acetate-induced protein 1), Aven (Cell death regulator Aven) and Myc (Oncogene Myc).^15^ The dysfunctional mitochondrial results in loss of inner mitochondrial membrane potential, hyperproduction of superoxide ions, disturbance in mitochondrial biogenesis, the outflow of matrix calcium glutathione and release of membrane proteins,^14,15^ hold promising potential for cancer therapeutic strategies via induction of apoptosis in cancer cells which are discussed later in this review.

### Execution pathway

Both extrinsic and intrinsic pathways converge at the same point (execution phase). Execution phase refers to the final pathway of apoptosis.^54^ Caspase-8, and 9 are initiator caspases while caspase-3, caspase-6 and caspase-7, Caspase-10, CAD (Caspase-activated DNAse) and PARP (Poly (ADP-ribose) polymerase) are classified as effector or executioner caspases.^55,56^ Initiator caspases activated as a result of autocleavage, which further activates executioner caspases which later proteolyze some substrates leading to apoptosis. They possess long pro-domains that connect large adapter molecules promoting multimerization and result in other caspases activation. However, effector caspases possess short pro-domains which execute apoptosis when activated by initiator caspases. Executioner caspases activate cytoplasmic endonuclease which causes chromatin condensation, the formation of cytoplasmic blebs and apoptotic bodies. Caspases regulate apoptotic cell death via cleavage of numerous target proteins.^57,58^ The pathway begins with the activation of execution caspases which further activates cytoplasmic endonuclease. Cytoplasmic endonuclease degrades nuclear material, and proteases followed by the degradation of nuclear and cytoskeletal proteins. Among all executioner caspases, caspase-3 is the most important, and any of the initiator caspases can activate it. Endonuclease CAD is activated explicitly by caspase-3, which causes degradation of chromosomal DNA within nuclei and condensation of chromatin. Execution caspases play an essential role in the cytoskeletal reorganization and formation of cytoplasmic blebs and apoptotic bodies.^15,46^

### Therapeutic targets for targeting death receptors: extrinsic pathway

**Figure 4:**
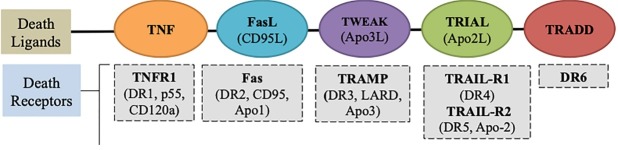
Death ligands and their receptors Death ligands and their receptors comprise of Tumor necrosis factor (TNF)- Tumor necrosis factor receptor (TNFR1), Fas ligand (FasL)/CD95L-Fas, TNF-related weak inducer of apoptosis (TWEAK/Apo3L)- TNF receptor–related apoptosis-mediating protein (TRAMP), TNF related apoptosis inducing 13 ligand (TRIAL/Apo2L)- TNF related apoptosis inducing ligand receptor (TRAIL-R1) and Tumor necrosis factor receptor type 1-associated death domain (TRADD)- Death receptor (DR6). TNF-related apoptosis-inducing ligand (TRAIL). This ligand-receptor binding interaction activate the cytoplasmic domain, consequently recruit and activates extrinsic pathway initiator caspases such as caspase -8 and caspase 10.

DRs are associates of TNF superfamily and initiate apoptotic signals when similar death ligands bind to the particular cell surface of DRs.^59^ Death ligands and their respective receptors comprise of TNF-TNFR1, FasL/CD95L-Fas, TWEAK(Apo3L)-TRAMP, TRIAL(Apo2L)-TRAIL-R1 and TRADD-DR6 as shown in Figure 4. The DRs well characterized by cysteine-rich extracellular domains and intracellular cytoplasmic sequence called as DD. This ligand-receptor binding leads to the activation in the cytoplasmic domain, accumulation of receptor and employment of adaptor proteins through the interaction between the adoptors and DD of receptors. Which consequently recruit and activates extrinsic pathway initiator caspases such as caspase -8 and caspase 10.^60,61^

DRs play a significant role in the extrinsic apoptotic pathway and therefore emerged as a potential cancer therapeutic target. A variety of agents proposed in order to stimulate the apoptotic function of DRs and ligands in the extrinsic pathway, such as DNA damaging chemotherapeutic agents, histone deacetylase (HDAC) inhibitors, proteasome inhibitors, cyclooxygenase-2 inhibitors and a number of antibodies which target the DR. Similarly, DNA damaging chemotherapeutic agents targeting Fas (DR2) expression include cisplatin, mitomycin, methotrexate, mitoxantrone, doxorubicin, and bleomycin. Moreover, etoposide, Ara-C, and camptosar (CPT-11) are used to target TRIAL-R1 (DR4) and TRIAL-R2 (DR5), thereby stimulating their expression.^62^ A number of HDAC inhibitors like suberoylanilide hydroxamic acid (SAHA), trichostatin A (TSA), LAQ824 (a cinnamic acid hydroxamate), m-carboxycinnamic acid bishydroxamide (CBHA) and MS-275 used in order to stimulate the expression of TRIAL-R1 and TRIAL-R2.^63,64^ MG132 is an example of proteasome inhibitors which effectively enhance the expression of TRIAL-R2 (DR5) while PS-341 is another proteasome inhibitor which promotes the expression of TRIAL-R1/2 without affecting the expression of pro-apoptotic Bcl-2 family proteins, cFLIP and caspases.^65,66^ A variety of antibodies have been utilized to target DRs (TRIAL), Apo2L/TRAIL showed as a potential cancer therapeutic agent, induce apoptosis in cancer cells via its two major cell DRs TRAIL-R1 and TRAIL-R2.^67,68^ They have specifically shown to expressed at higher levels in solid tumors.^69^ Owing to the ability of TRAIL receptors of inducing cell death specifically in cancer cells, agonistic antibodies against TRAIL receptors have been developed and demonstrated to trigger apoptosis in a number of cancer cells.^70^ various agonists targeting DRs in clinical trials. Dulanermin targets both TRIAL-R1/2 for colorectal cancer CRC and non-small cell lung cancer NSCLC, Mapatumumab target TRIAL-R1 for advanced solid tumors and NSCLC, PR095780 for Advanced solid tumors, NHL in I and II phase.^71-76^ Lexatumumab (HGS-ETR2) and Conatumumab (AMG-655) target TRIAL-R2 for Advanced solid tumors in I phase.^77-80^ Despite all the success of TRAIL targeted cancer therapy, TRAIL resistance is a common hindrance in TRAIL-based therapy that restricts the efficacy of these drugs.^81^

cFLIP (cellular FLICE-like) inhibitory protein is a crucial anti-apoptotic regulator that suppresses cell death induced by the DRs such as Fas-L, TNF-α and TRAIL.^80^ cFLIP (27 kDa protein) comprises two DEDs which can inhibit FADD and recruited procaspase-8 interaction by binding to DED of FADD and consequently results in the inactivation of caspase-8. A variety of drugs have been developed to trigger the activation of caspases. Such as apoptin (caspase inducing agent) have been utilized for the activation of caspases-3 and caspase-7. Apoptosis facilitates the execution of apoptosis by causing DNA damage and also aid in the release of cytochrome c from mitochondria.^83,84^ Moreover, targeting caspase-8 can result in therapeutic effect by utilizing decitabine (5-aza-2´deoxycytidine) which is a cytosine nucleoside analog and endorse demethylation by constraining DNA methyltransferase covalent binding especially in tumors suffering from hypermethylation of caspase-8 promotors, thus restoring the expression of caspase-8.^85^ Also, gene therapy to induce caspase based apoptosis has adopted by utilizing the genes that encode for inducer, mediator or executioner of apoptosis and also through suppressing the anti-apoptotic gene expression. Selective gene delivery, particular gene expression, and genetic modification by secreting target proteins are some of the strategies adopted to date in apoptosis-based cancer gene therapy.^86^ The three isoforms of cFLIP in humans include c-FLIPL (long), c-FLIPS (short), and c-FLIPR (splice).^60^ Generally, higher concentration or enhanced expression of c-FLIPS and c-FLIPL results in the anti-apoptotic function of cFLIP.^87^ Increased expression of cFLIP observed in different types of cancer leading to enhanced cancer progression.^88^ Similarly, the reduced expression or down-regulation of cFLIP can inhibit the proliferation of cancer cells and aid in the induction of apoptosis mediated by the DRs and the intrinsic mitochondrial pathway.^89^ Thus, cFLIP can play an essential role in cancer therapy, specifically if used with TRAIL or conventional chemotherapy.^63^ Moreover, even though c-FLIPL can perform a dual role in apoptosis (pro- and anti-apoptotic), typically the primary function of cFLIPL have been recognized as an anti-apoptotic regulator of apoptosis in cancer.^90^

Various approaches that have taken to reduce or suppress the anti-apoptotic function of cFLIP involve the use of siRNA (small interfering RNA), use of many small molecules and agents that down-regulate cFLIP.^91^ Specifically, siRNA inhibits the expression of c-FLIP and prepare cancer cells to be receptive or sensitize for TRAIL, FASL, and chemotherapeutic agents that induce apoptosis. However, the use of siRNA in vivo involves some restrictions. Besides, the employment of siRNA to inhibit cFLIP depends on the safe delivery of siRNA.^92^ Many small molecules have been utilized to reduce mRNA and protein intensities of c-FLIPL, however, due to the significantly ordinary homology of cFLIP and caspase-8, use of small molecules for the inhibition of its activity is very challenging.^88^ Other than utilizing small molecules, different classes of agents have recognized that down-regulate c-FLIP expression by affecting cFLIP transcription, translation and degrading cFLIP.^91,93^ These agents include conventional chemotherapeutic drugs, DNA damaging agents and HDAC inhibitors. The conventional chemotherapeutic drugs and DNA damaging agents are cisplatin, doxorubicin, camptothecin, 9-nitrocamptothecin, and oxaliplatin. HDAC inhibitors include SAHA and the inhibitors of MEK1/2, PKC, and PI3K.^31,86^ DR agonists represent an effective therapeutics that mainly target apoptosis. Further, clinical trials of these agents showed the safety of the approach and apoptotic cell death. Forthcoming data from recent trials will also help to demonstrate their clinical activity in different tumor types alone and combinations. Although understanding the mechanism of the TRAIL pathway, studying various factors that might halt response, win over the mechanisms of tumor-cell resistance, and get benefit from these therapies.

### Therapeutic targets for targeting anti-apoptotic protein of Bcl-2 family: intrinsic pathway

Bcl-2 family proteins that comprise of pro- and anti-apoptotic proteins are known to play an essential role in the regulation of intrinsic pathway of apoptosis.^94^ The categorization of Bcl-2 family proteins based on the existence of shared blocks of sequence homology, named as Bcl-2 homology (BH). The equilibrium between pro- and anti-apoptotic Bcl-2 family proteins is an essential element for the initiation of mitochondrial outer membrane permeabilization (MOMP).^95^ Bcl-2, Bcl-XL, and Mcl-1 are anti-apoptotic proteins and their role is to prevent the release of cytochrome c and maintain mitochondrial integrity while Bax, Bak, Bad, and Bok are pro-apoptotic proteins of Bcl-2 family which allow the release of cytochrome c from the mitochondrial intermembrane space into the cytosol to promote the induction of apoptosis eventually aid in cancer therapeutics.^51,52^ Up-regulation of pro-apoptotic Bcl-2 proteins and down-regulation of anti-apoptotic Bcl-2 effectively linked to the mechanism of cell death. For example, the ratio between pr-apoptotic (Bax) and anti-apoptotic (Bcl-2) proteins is generally used to determine the fate of the cell.^96^ Inactivation of pro-apoptotic proteins with multidomain (Bax and Bak) is a crucial feature of carcinogenesis.^97^ Similarly, elevated levels of anti-apoptotic proteins multidomain (BCL-2, BCL-xL, BCL-w, Bfl-1, and Mcl-1) encourage the deregulation of apoptosis in cancer cells and also aid cancer cells to become resistant to immune-surveillance.^98^ However, single proapoptotic domain BH3, i.e., BID, BIM, BAD, PUMA (p53 upregulated controller of apoptosis) and NOXA play a primary role in regulating and triggering apoptosis act as sensitizer and serve as an excellent therapeutic target. The overexpression of anti-apoptotic Bcl-2 family members or underexpression of pro-apoptotic Bcl-2 family members usually associated with chemoresistance. Further, BCL-2 over-expression has found in acute myeloid leukemia, chronic lymphocytic leukemia (CLL), non-Hodgkin’s lymphoma (NHL), myeloma, melanoma and hepatocellular, lung, breast, prostate carcinomas.^99-101^

Potential therapeutic agents with improved efficacy have been developed to target the down-regulation of anti-apoptotic and up-regulating pro-apoptotic Bcl-2 protein.^102^ Effective strategies have been adopted to inhibit the anti-apoptotic effects of Bcl-2 family, which include: using antisense oligonucleotides, development of small drug molecules and inhibit the gene transcription.^96^ An example of novel Bcl-2 antisense is Oblimersen Sodium (G3139, Genasense), 18-base antisense phosphorothioate oligonucleotide used in I and II phase of clinical trials in advance solid cancer lymphoma.^103,104^ It has also tested in combination with other anticancer agents, such as Oblimersen with rituximab used for NHL in II phase, Oblimersen with dacarbazine for myeloma in III phase, Oblimersen with docetaxel for castration-resistant prostate cancer (CRPC) and breast cancer in II and I phase, non-small-cell lung carcinoma (NSCLC) or small-cell lung carcinoma (SCLC) in III phase and HRPCa (EORTC) in II phase.^105-109^

HDACs are attractive therapeutic targets in cancer and inflammatory diseases.^110^ A significant controllers of gene expression work enzymatically in removing the acetyl group from histones proteins.^111-113^ Genetic knock-down has been shown the role of HDACs induce apoptosis and cell cycle arrest in different tumor types, such as colon, lung, breast carcinomas and acute promyelocytic leukemia, highlighting its activity as a critical indicator of survival in cancer cells.^114^ Further, over-expression of HDACs has been linked to various critical events of tumorigenesis, includes epigenetic repression of CDKN1A (encoding the cyclin-dependent kinase inhibitor p21) tumor suppressor gene and essential genes, like breast cancer 1, early onset BRCA1 and ataxia telangiectasia and Rad 3 related (ATR).^94,95^ The Sodium butyrate is small molecule Inhibitors (HDAC inhibitor) involved in gene expression alteration in regulation of proapoptotic proteins. Another molecule Flavopiridol (cyclin-dependent kinase inhibitor) and are used to down-regulate the Bcl-2 and Bcl-xl, Mcl-1 expression respectively. Also, Fenretinide, which is a synthetic cytotoxic retinoid, acts by down-regulating the activity of Bcl-2 and Mcl-1 without altering the expression of pro-apoptotic protein Bax.^40^ Many HDACi have entered phase I to III clinical trials such as, CHR-3996 used for a Refractory solid tumor in phase I.^115^ Another inhibitory agent Panobinostat (LBH589) used for Relapsed or refractory NHL and advanced solid tumors and Panobinostat (LBH589) along with melphalan for Relapsed or multiple refractory myelomas in I and II phase.^116-118^

Natural and synthetic small-molecules BH3-mimicking agents have successfully antagonized antiapoptotic Bcl-2 protein family members, such as Obatoclax, Gossypol, ATB-263 and ATB-199.^119-122^ All BH3 proteins composed of the single domain called α-helical BH3 domain has been demonstrated to play a crucial role in cancer therapy.^123-125^ The BH-3 mimetics have developed which precisely bind to the hydrophobic groove (which facilitate the binding between pro- and anti-apoptotic proteins), and by this means they oppose the function of anti-apoptotic Bcl-2 family proteins.^95^ Bcl-2 antisense causes the down-regulation of Bcl-2 proteins by affecting the corresponding mRNA. Potential BH-3 mimetic drugs such as, Obatoclax Mesylate (GX15- 070MS) for SCLC and myelofibrosis and Gossypol/ AT-101 for Metastatic breast cancer and CRPC in I & II phase which inhibits Bcl-2, Bcl-xL, and Mcl- 1 expression. ATB-263 and ATB-199 inhibit/block Bcl-2 for Advanced hematological cancers and CLL 73,74 and MIM1 which inhibits Mcl-1 in clinical trials.^126^

### Therapeutic agents for targeting the Tumor suppressor protein: p53

Tumor suppressor gene p53 is the primary entities involved in carcinogenesis plays an essential part in cancer concerning both cell cycle arrest, and apoptosis.^127^ Tumor suppressor genes are responsible for controlling DNA repair and cell division. dysfunctional tumor suppressor genes could result in uncontrolled multiplication of cells leading to cancer. Many aspects like chemicals, ionizing radiation, and viruses can cause alterations in proto-oncogenes and tumor suppressor genes.^127-129^ The expression of p53 is deficient in normal cells under non-stressed conditions. However, p53 can be activated by any stress stimuli; DNA damage or in the response of oncogene activation. Extra and intracellular stress signals change latent p53 to an active form and encourage p53 to accumulate in a cell nucleus. The stability of activated p53 regulated through various post-translational chemical modifications like phosphorylation, acetylation, and methylation.^130,131^ The fundamental role of p53 is its capability to induce apoptosis by transcription-dependent and transcription-independent manner. p53 performs its function by transcription activation of pro-apoptotic Bcl-2 family proteins and transcription suppression of anti-apoptotic Bcl-2 family proteins. Moreover, it can directly interact with Bax that successively stimulates the release of cyt C via MOMP and aid in the induction of apoptosis.^132^

Different small molecules MDM2 inhibitors that have been developed to trigger wild-type p53 activity, such as Nultlin-3, MI-219, and RITA. The role of Nultlin-3 and MI-219 is to prevent the interaction of MDM2 and p53, activating p53 signaling and suppressing the tumor growth.^133-135^ However, the pharmacological action of Nutlin-3 is via both the transcription-dependent and - independent p53 apoptotic pathways.^32,136,137^ Nutlin-3 has been shown to induce mitotic arrest rather than apoptosis mainly.^138^ MDM2 can also trigger, in response to low genotoxic damage, the downregulation of p53 apoptotic activator HIPK2.^139^ Interestingly, Nutlin-3 along with zinc ion inhibit the MDM2 ligase activity favoring HIPK2 stabilization results in an induction of p53 apoptotic activity.^140^ Similarly, co-treatment of Nutlin-3 and (ABT-737) Bcl-2 inhibitor has been shown to enhance the sensitivity to apoptosis of cancer cells greatly.^141^ Further, multi-target anticancer approach, inhibition of both MDM2 and Bcl-2 could be a positive tool in cancer treatment.^142^ Another reported small molecule MDM2 inhibitor is CP-31398, which increases the transcriptional activity of p53 in cells.^143^ Furthermore, three different types of p53 vaccines such as peptide-based vaccines, dendritic-cell based vaccines, and recombinant virus-based vaccines are undergoing clinical trials to assist in the induction of antitumor immune responses.^144-146^ Another research reveals a class of small molecules that reactivates the wild-type function of mutant p53 in so doing permit p53 to induce apoptotic cell death. PRIMA-1 and its analog APR-246 are examples of this class of small molecules and are undergoing preclinical and clinical trials (phase I) to functions as reactivating mutant p53.^147^

### Therapeutic agents for targeting Inhibitory apoptosis Proteins: IAPs

The IAPs is family of protein which functions as endogenous inhibitors of apoptosis. Elevated expression levels of IAPs were significantly resulting in improved cell survival, increased tumor growth and consequent metastasis. IAPs targeting strategy has become increasingly attractive to sensitize cancer cells towards various therapeutics such as chemotherapies, antibody based-therapies. Besides apoptosis, IAPs observed to play a part in necroptosis, immune regulation, chromosomal and cytoplasmic division.^148^ IAPs can inhibit both intrinsic and extrinsic pathway of apoptosis. The execution of DR-mediated extrinsic pathway and mitochondrial triggered a family of structurally diverse IAPs modulates intrinsic pathway; X-linked (XIAP), cellular (cIAP1, cIAP2), neuronal (NIAP), testis-specific (Ts-IAP), Bir-ubiquitin conjugating enzyme (BRUCE), Survivin and Livin. Structurally, IAPs are approximately 70 amino acids long and contain zinc finger BIR (Baculovirus IAP Repeat) domains that are responsible for deregulation properties of IAPs where they prevent the conversion of zymogenic (inactive) pro-caspases to active caspases.^149,150^ Over-expression of IAPs linked to increased chemo-resistance in several types of cancer.^151,152^ As a controlled expression of IAPs could encourage apoptotic cell death, different strategies have been adopted to inhibit IAPs, and these include: anti-sense facilitated interference of XIAP and survivin oligonucleotides and siRNA expression and inhibition of IAPs by SMAC mimetic compounds.^153-155^ XIAP inhibits both the extrinsic and intrinsic apoptotic pathways via direct inhibition of enzymes caspases and may be limited by its initiation of cell protective effects via NF-kB signaling and cIAP1/2 through proteasomal degradation or ubiquitination.^156^ Knockout strategies in cancer cells were highlighting their role in resistance to various anti-cancer therapies. For example, increased apoptosis suppressed tumorigenicity and re-sensitized was reported ovarian cancer cells to cisplatin therapy and in nude mice through shRNA mediated knockdown of XIAP.^157^ The successful results in acute myeloid leukemia patients undergoing therapy using antisense oligonucleotide AEG35156 that target XIAP in phase II trials.^158,159^ However, despite this initial success and confirmed on-target knockdown,^157^ a later trial failed to report a similarly improved outcome in patients with advanced pancreatic cancer.^160^ while gene silencing is an attractive prospect, its potential clinical relevance is limited by lower knockdown efficiency in patient samples, compared to those demonstrated in cell culture.^158^ and by the transient nature of XIAP repression.^160^ Still, strategies for RNAi remain important tools to dissect the mechanistic and functional role of IAPs in cancer.

**Figure 5:**
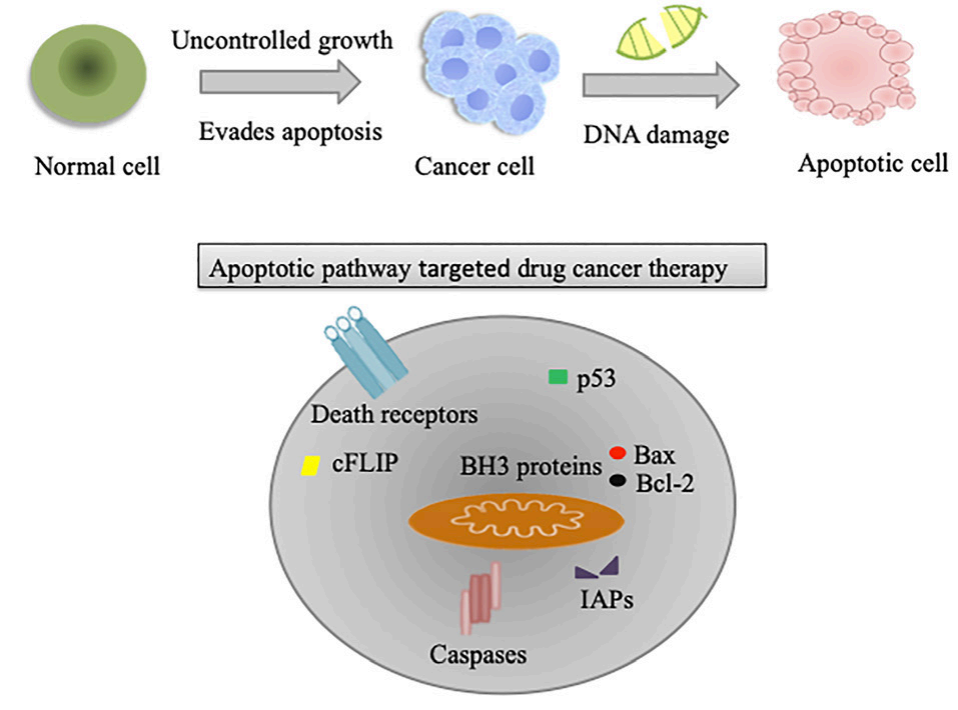
Apoptotic pathway targeted drug therapy Apoptotic pathways targeted strategies such as death receptors, antiapoptotic Bcl-2 family, IAPs, caspases and p53 showed positive results in previous studies need to be investigate more to get effective therapeutics.

SMAC mimetics release into the cytosol as a result of MOMP binds to the BIR domain cellular IAPs (cIAP1 and cIAP2) and XIAP, restoring the function of effector caspases by blocking inhibitory role of IAPs.^161^ To date, some inhibitors of IAP proteins have been developed these include: SH122, SH130, SM164, AZD5582, JP1201, AEG35156, LY2181308 and YM155.^151^ SMAC mimetic SH130 and SH122 target human prostate cancer cell line by inhibiting IAPs.^162,163^ AZD5582 and JP1201 are SMAC mimetics which target CLL and pancreatic cancer cell line respectively to enhance apoptosis by TRAIL.^164,165^ YM155 suppresses survivin expression and induce apoptosis in human cancer cell lines.^166,167^ AEG35156 and LY2181308 are antisense oligonucleotides and small siRNA molecules which targets survivin expression and also down-regulate XIAP.^168,169^ Various past researches demonstrated that SMAC mimetics, along with anticancer drugs and TRAIL remarkably enhance apoptotic cell death in several cancer cell types *in vitro*, such as T98G glioblastoma cells, HeLa cells, and lung adenocarcinomas.^170-172^ Further, the SMAC mimetics role as sensitizer enhance the sensitivity of various agents, such as paclitaxel, etoposide, and doxorubicin in MCF-7 breast cancer cells.^173^ Various apoptotic pathways targeted strategies as shown in Figure 5, such as DRs, antiapoptotic Bcl-2 family, IAPs, caspases and p53 need intense focus in development of effective drug therapy. Studies suggested that IAPs may be useful as single agents in cancer also usefulness antagonists in combination with alternative cancer drugs. As further research progress, the better improvements in understanding of therapeutic design which may enhance DR, Bcl-2, IAP and p53 mediated cell death in single as well as combined treatment.

## Conclusion

Targeting apoptosis is a new standard in cancer drug development: a significant regulatory mechanism inside cells, a cellular process that is tightly regulated by various membrane-bound and freely available cytoskeleton molecules. Apoptosis is a fundamental regulatory mechanism of normal cells, any dysregulation in apoptosis could trigger the uncontrol multiplication of cells. This regulatory process characterized by a series of specific morphological changes along with biochemical features which involve extrinsic and intrinsic pathways via a different protein that plays a crucial role overall. Therefore, a detailed mechanistic understanding of the apoptotic signaling pathways required for the development of effective cancer therapeutics. The up-regulation of apoptotic pathways via activation of pro-apoptotic pathway proteins (initiator and effector caspases, box, bak, bad and bok along with inhibition of cFLIP and sensitize DRs to trigger apoptotic pathway). The understanding of apoptotic pathways needs intense effort for the development of new approaches to drug discovery and therapy. However, some apoptotic pathways proteins which induce apoptosis selected as a target for drugs are in clinical trials. Positive results of antibodies along with recombinant TRAIL specifically target the DRs in clinical trials against a range of solid tumors. However, much understanding of evading apoptosis in cancer cells is needed to get the positive results in maturing clinical data. The novel agents along with combined apoptotic inhibitors strategy show significant synergistic effects and being in the current study.

## Ethical Issue

Not applicable.

## Conflict of Interest

The authors declare no conflict of interest.
